# Prognostic impact of tumor-associated macrophage infiltration in non-small cell lung cancer: A systemic review and meta-analysis

**DOI:** 10.18632/oncotarget.9079

**Published:** 2016-04-28

**Authors:** Jiandong Mei, Zhilan Xiao, Chenglin Guo, Qiang Pu, Lin Ma, Chengwu Liu, Feng Lin, Hu Liao, Zongbing You, Lunxu Liu

**Affiliations:** ^1^ Department of Thoracic Surgery, West China Hospital, Sichuan University, Chengdu 610041, China; ^2^ Western China Collaborative Innovation Center for Early Diagnosis and Multidisciplinary Therapy of Lung Cancer, Sichuan University, Chengdu 610041, China; ^3^ Department of Structural and Cellular Biology, Tulane University, New Orleans, LA 70112, USA

**Keywords:** clinicopathological characteristics, non-small cell lung cancer (NSCLC), prognosis, tumor-associated macrophages (TAMs)

## Abstract

Tumor-associated macrophages (TAMs) are important components of cancer microenvironment. In the present study, we searched PubMed, Embase, Cochrane library and Web of Science to perform a meta-analysis of 20 studies including a total of 2,572 non-small cell lung cancer (NSCLC) patients, in order to determine the association between TAMs and NSCLC prognosis. The combined hazard ratio (HR) of 9 studies showed that the density of total CD68^+^ TAMs in the tumor islet and stroma was not associated with overall survival (OS) of the patients. However, the pooled HR of 4 studies showed that high density of CD68^+^ TAMs in the tumor islet predicted better OS, while the pooled HR of 6 studies showed that high density of CD68^+^ TAMs in the tumor stroma was associated with poor OS. A high islet/stroma ratio of CD68^+^ TAMs was associated with better OS. A high density of M1 TAMs in the tumor islet was associated with better OS, while a high density of M2 TAMs in the tumor stroma predicted poor OS. These findings suggest that, although the density of total CD68^+^ TAMs is not associated with OS, the localization and M1/M2 polarization of TAMs are potential prognostic predictors of NSCLC.

## INTRODUCTION

Lung cancer is the most common cause of cancer-related deaths in both men and women worldwide [1]. New therapeutic modalities, such as minimally invasive surgery and targeted therapy, have been introduced to the treatment of lung cancer during the past decades. However, the overall 5-year survival of lung cancer patients has been improved very little, especially in the advanced diseases [2]. Immunotherapy has brought up new options for lung cancer patients, including blockade of immune checkpoints like cytotoxic T-lymphocyte-associated protein 4 (CTLA-4) and programmed cell death 1 (PD-1) [3, 4]. However, due to the complexity of the tumor microenvironment and the interactions among each tumor components, development of new combinational therapies targeting different mechanisms involved in tumor progression may open a new era of lung cancer therapy.

Tumor-associated macrophages (TAMs) are an important component of the tumor microenvironment [5, 6]. This group of immune cells function as immune regulators in the tumor microenvironment and are potential targets of cancer immunotherapy [7]. TAMs may have both anti- and pro-tumor effects due to two distinctly different polarizations, i.e., M1 (also known as classically activated) and M2 (alternatively activated) TAMs [8]. Mills et al. recently described these two types of activated macrophages as ‘Inhibit’ type (M1) and ‘Heal’ type (M2) due to their stimulation of Th1 or Th2 type responses [9, 10]. The M1 polarization is known to be induced by interferon-γ, lipopolysaccharide (LPS), tumor necrosis factor α (TNF-α), and granulocyte-macrophage colony-stimulating factor (GM-CSF) [8, 10, 11]. M1 macrophages function to promote Th1 responses with microbicidal and tumoricidal effects [8, 10, 11]. The M2 macrophages are known to be activated by interleukin-4 (IL-4), IL-10, IL- 13, and prostaglandin E2 (PGE2) [11–13]. Activation of M2 macrophages induces Th2 responses and promotes tissue repair and remodeling, angiogenesis, and immune suppression, as well as tumor progression [11, 13, 14].

The dual roles of TAMs in tumor progression have been supported by both *in vitro* and *in vivo* studies using different tumor models. However, the role of TAMs in lung cancer progression remains controversial due to the discrepancies among the previous studies on TAM infiltration and lung cancer prognosis. A variety of markers (including CD68, CD163, CD204, HLA-DR, etc.) were used to identify different types of TAMs and the micro-distribution (in the tumor islet, stroma, or both) of TAMs in the tumor tissues [15–19]. However, some technical pitfalls have compromised the conclusions drawn from the previous studies, such as small sample size that limited the statistical power in revealing the implications of TAMs on the clinicopathological characteristics and prognosis. Therefore, we conducted the present meta-analysis to evaluate the role of different types and distribution of TAMs in the tumor microenvironment of non-small cell lung cancer (NSCLC), through pooling data from 20 eligible studies.

## RESULTS

### Characteristics of studies

**Figure 1:**
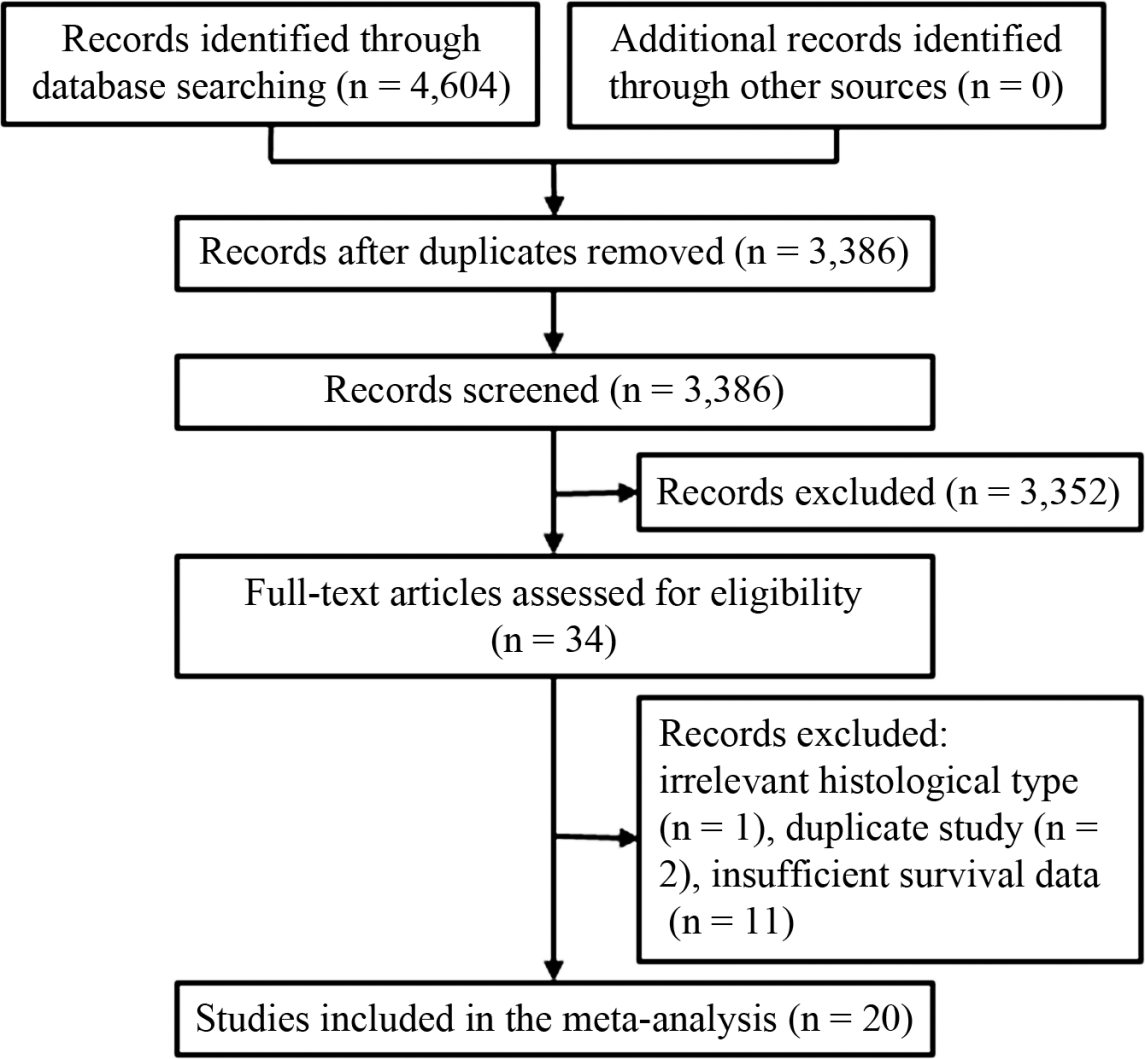
Flow chart for the selection of studies A total of 20 studies were selected to determine the association between TAM density and lung cancer prognosis.

A total of 4,604 records were identified from different databases during primary search. These records were screened and irrelevant results were excluded as shown in the search flow diagram (Figure 1). Full-texts of 34 candidate studies were carefully reviewed and 14 of them were excluded. The remaining 20 original reports [15–19, 22–36] published between 1999 and 2014 were included in this meta-analysis. Detailed information of these studies is listed in Table 1. The mean NOS score of these included studies was 7.95.

**Table 1:** Characteristics of eligible studies

Author	Year	Region	Study Period	Cases	Type	Stage	Markers	Group	TAM distribution				Polarization		Outcome		NOS Score
									I+S	I	S	I/S	M1	M2	DFS	OS	
Pei [15]	2014	China	2003–2008	417	NCLC	I–III	CD68	− vs. +	√	-	-	-	-	-	√	√	9
Li [22]	2014	China	2007.1–2008.6	130	ADC, SCC	I–IV	CD163	< M vs. > M	-	-	√	-	-	-	-	√	8
Feng [23]	2014	Taiwan	2005–2008	28	NSCLC	IIIA–N2	CD68	< M vs. > M	√	-	-	√	-	-	√	√	8
Carus [16]	2013	Denmark	2003–2006	335	NSCLC	I–IIIA	CD163	< M vs. > M	-	√	√	-	-	-	-	√	9
Hirayama [17]	2012	Japan	2000–2006	208	SCC	I–IIIA	CD204	< M vs. > M	-	-	√	-	-	-	√	√	9
Paola [24]	2012	Brazil	N/A	65	NSCLC	I–IIIA	CD68	≤ 4.5% vs. > 4.5%	√	-	-	-	-	-	-	√	5
Zhang [25]	2011	China	2003–2006	65	ADC	I–IV	CD68+iNOS/CD206	< M vs. > M	√	-	-	-	√	√	-	√	9
Ohtaki [26]	2010	Japan	1996.1–1998.3	170	ADC	I–IIIA	CD68/CD204	< M vs. > M	-	-	√	-	-	-	-	√	8
Ma [27]	2010	China	1999.6–2001.8	100	NSCLC	I–IV	CD68+HLA–DR/CD163	< M vs. > M	√	√	√	-	√	√	-	√	8
Dai [18]	2010	China	1999.8–2001.8	99	NSCLC	I–IV	CD68	< M vs. > M	√	√	√	√	-	-	-	√	9
Ohri [19]	2009	UK	1991–1994, 1999	40	NSCLC	I–IV	CD68+HLA–DR/CD163	< M vs. > M	-	√	√	-	√	√	-	√	8
Al-Shibli [28]	2009	Norway	1990–2004	333	NSCLC	I–IIIA	CD68	< 25% vs. > 25%	-	-	√	-	-	-	-	√	8
Kim [29]	2008	Korea	1997–1998	144	NSCLC	I–IV	CD68	< M vs. > M	√	√	√	-	-	-	-	√	9
Kawai [30]	2008	Japan	1996–2004	199	NSCLC	IV*	CD68	< M vs. ≥ M	-	√	√	√	-	-	-	√	8
Ho [31]	2008	Taiwan	1996.9–1998.9	68	NSCLC	I–III	TREM1	< M vs. > M	√	-	-	-	-	-	√	√	7
Welsh [32]	2005	UK	1991–1994, 1999	118	NSCLC	I–IV	CD68	< M vs. > M	-	√	√	√	-	-	-	√	9
Chen [33]	2005	Taiwan	1994.9–1996.9	41	ADC, SCC	I–IV	CD68	< M vs. > M	-	√	-	-	-	-	√	-	7
Chen [34]	2003	Taiwan	1994.5–1994.12	35	SCC, ADC	I–IIIA	CD68	< M vs. > M	√	-	-	-	-	-	-	√	7
Takanami [35]	1999	Japan	1986–1992	113	ADC	I–IV	CD68	≤ Mean vs. > Mean	√	-	-	-	-	-	-	√	7
Eerola [36]	1999	Finland	1978–1995	38	LCLC	I–III	CD68	< M vs. > M	√	-	-	-	-	-	-	√	7

ADC = Adenocarcinoma, DFS = Disease free survival, LCLC = Large cell lung cancer, I = Islet, M = Medium, NOS = Newcastle-Ottawa Scale, NSCLC = Non-small cell lung cancer, OS = Overall survival, S = Stroma, SCC = Squamous cell carcinoma, TAM = Tumor-associated macrophage, UK = United Kingdom

Among the included studies, some patients were enrolled twice for different research purposes in different publications, including the studies by Dai et al. [18] and Ma et al. [27], Welsh et al. [32] and Ohri et al. [19], and Chen's two studies [33, 34]. As a result, a total of 2,572 patients were studied in all 20 included publications. Large cell lung cancer (LCLC) [36] and lung squamous cell carcinoma (SCC) [17] were the only histologic type in each of these studies, respectively. Three studies investigated lung adenocarcinoma (ADC) [25, 26, 35]. Both ADC and SCC patients were included in the remaining studies (Table 1).

CD68 was a common monocyte/macrophage marker and was used as TAM marker in 16 studies, including 4 studies of CD68 in combination with other specific macrophage markers. Double immunohistochemical staining was applied to estimate the prognostic role of different TAM polarization and survival in 3 studies, while the other studies used single immunohistochemical staining. M1 TAMs were labeled as CD68^+^HLA-DR^+^ cells in 2 studies and as CD68^+^iNOS^+^ cells in a third study. M2 TAMs were indicated as CD68^+^CD163^+^ cells in 2 studies and as CD68^+^CD206^+^ cells in a third study. There were 2 publications studying CD163^+^ and another 2 articles studying CD204^+^ TAMs, including one study that estimated both CD68^+^ TAMs and CD204^+^ M2 TAMs simultaneously. The role of CD68^+^ TAMs in both tumor islet and stroma on overall survival (OS) was studied in 9 articles. The tumor islet and stromal CD68^+^ TAM densities were reported in 4 and 5 articles, respectively.

### TAM density in the tumor islet + stroma (I+S) and survival

**Figure 2:**
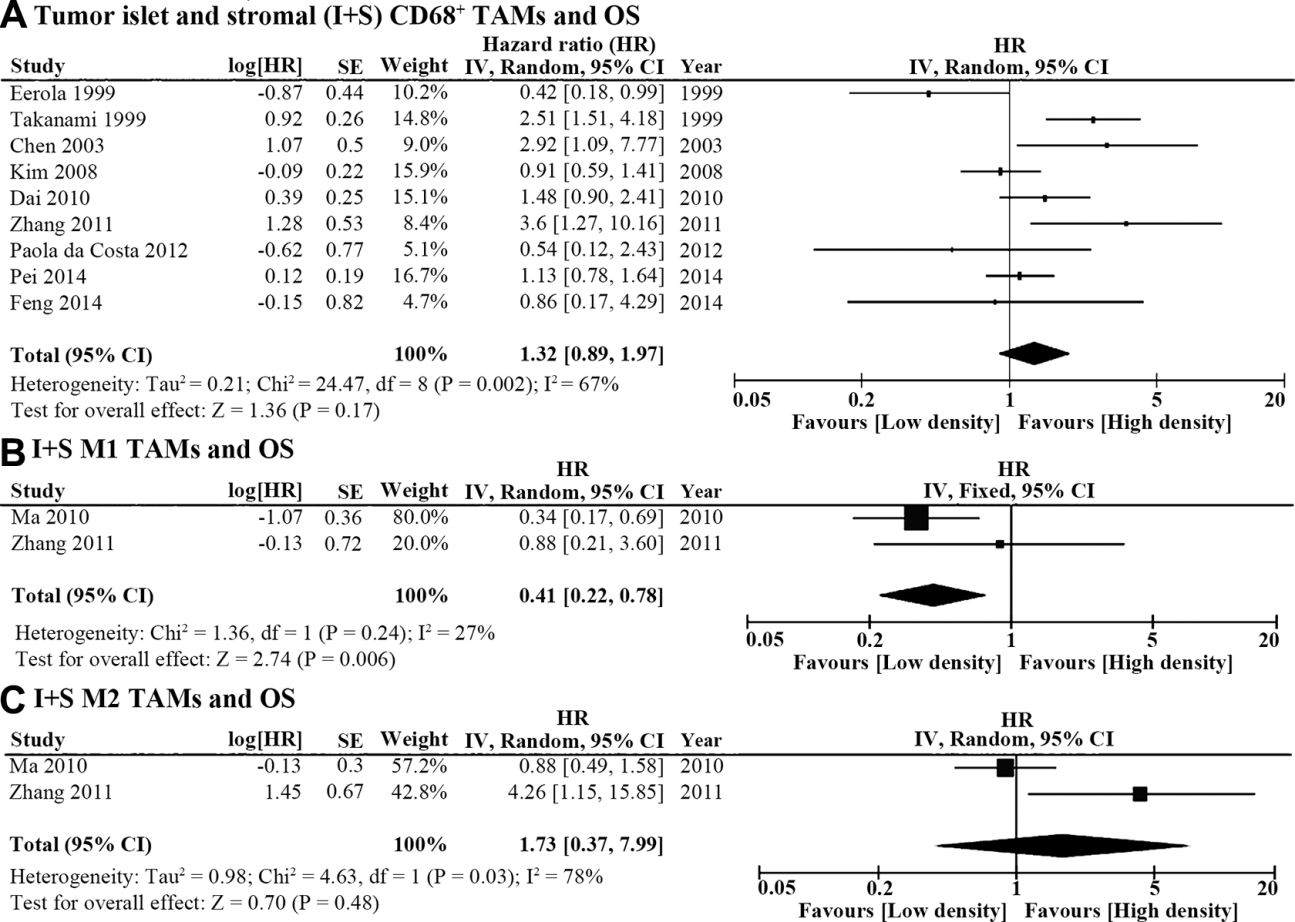
Forest plot of HR for TAM density in the tumor islet and stroma (I+S) and overall survival (OS) (**A**) The pooled HR of 9 studies showed that I+S CD68^+^ TAM density was not associated with OS (high CD68^+^ TAM density vs. low CD68^+^ TAM density, HR = 1.32, 95% CI = 0.89 ~ 1.97; *P* = 0.17; I^2^ = 67%, *P* = 0.002). (**B**) High I+S M1 TAM density predicted better OS (HR = 0.41, 95% CI = 0.22 ~ 0.78, *P* = 0.006; I^2^ = 27%, *P* = 0.24). C. I+S M2 TAM density was not associated with OS of lung cancer patients (HR = 1.73, 95% CI = 0.37 ~ 7.99, *P* = 0.48; I^2^ = 78%, *P* = 0.03).

Among the 20 included studies, 9 reported the relationship between CD68^+^ TAMs (I+S) and OS. The pooled HR of these 9 studies showed that CD68^+^ TAM infiltration was not associated with OS (high CD68^+^ TAM density vs. low CD68^+^ TAM density, HR = 1.32, 95% CI = 0.89 ~ 1.97; *P* = 0.17; I^2^ = 67%, *P* = 0.002; Figure 2A). The patients in 8 out of these 9 studies were grouped according to the count of CD68^+^ TAMs for high or low macrophage infiltration, while the study by Pei et al. [15] used tissue microarray and grouped patients as CD68 positive versus negative staining. Nevertheless, the conclusion remains unchanged after eliminating Pei's study. The pooled HR was 1.35 (95% CI = 0.82 ~ 2.22, *P* = 0.23; I^2^ = 70%, *P* = 0.001) in the remaining 8 studies. There were 2 studies focused on the I+S CD68^+^ TAM density and DFS [15, 23]. Another study used the protein named triggering receptor expressed on myeloid cells-1 (TREM-1) as a marker for TAMs and reported that TREM-1 was expressed only by CD68^+^ TAMs in lung cancer tissue [31]. The pooled HR of these 3 studies [15, 23, 31] showed no association between the I+S TAM density and DFS (HR = 2.21, 95% CI = 0.82 ~ 5.98, *P* = 0.12; I^2^ = 77%, *P* = 0.01).

In addition, we also assessed the association between OS and M1 or M2 polarization using the data reported in two studies [25, 27] that employed double immunohistochemical staining to identify M1 and M2 TAMs. Ma et al. marked M1 TAMs as CD68^+^HLA-DR^+^ cells and M2 TAMs as CD68^+^CD163^+^ cells [27]. Zhang et al. labeled M1 as CD68^+^iNOS^+^ cells and M2 TAMs as CD68^+^CD206^+^ cells [25]. The pooled HR of the two studies revealed that high M1 density in lung cancer tissues predicted better OS (HR = 0.41, 95% CI = 0.22 ~ 0.78, *P* = 0.006; I^2^ = 27%, *P* = 0.24; Figure 2B), while the density of M2 TAM infiltration was not associated with OS in lung cancer patients (HR = 1.73, 95% CI = 0.37 ~ 7.99, *P* = 0.48; *I*^2^ = 78%, *P* = 0.03; Figure 2C).

### Islet TAM density and OS

**Figure 3:**
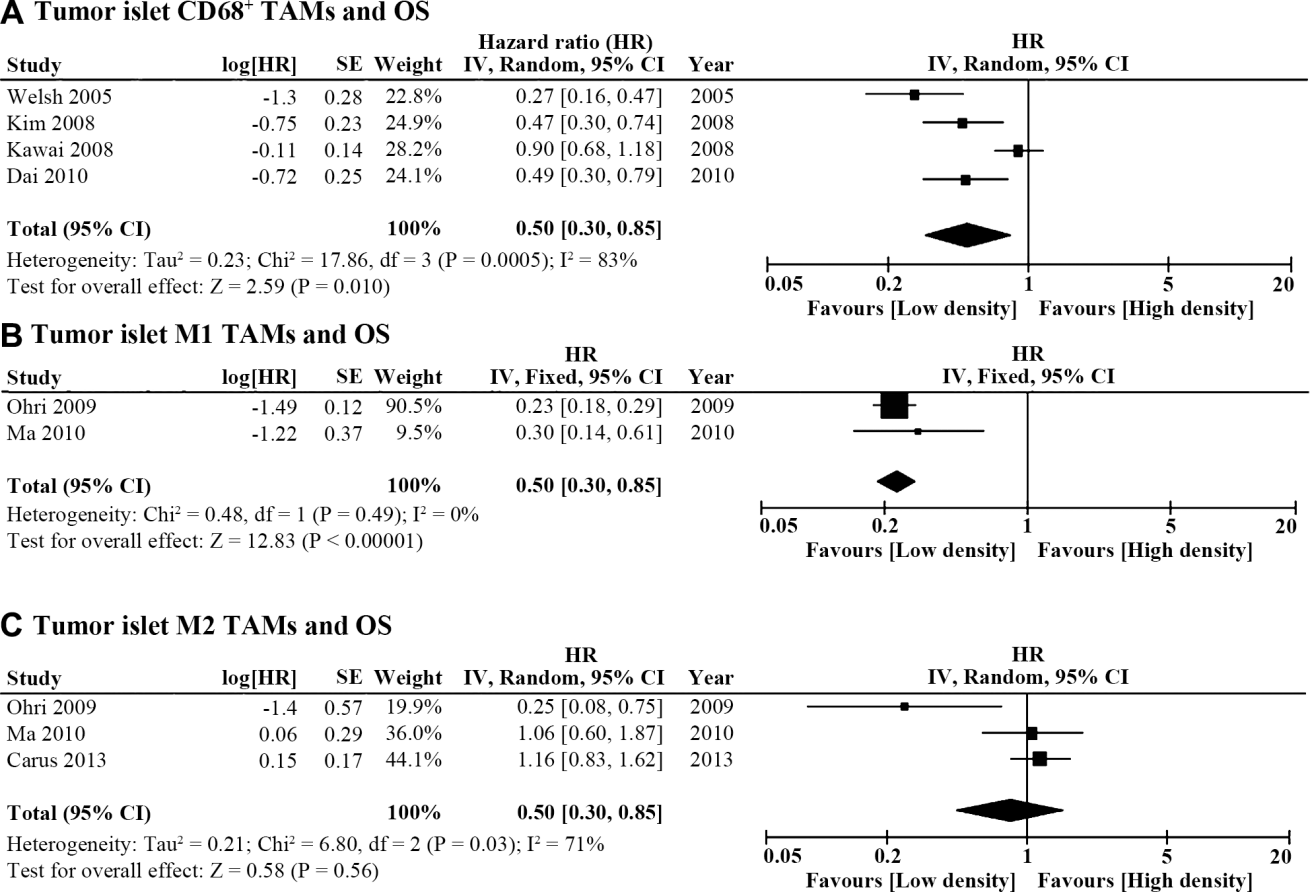
Forest plot of HR for TAM density in the tumor islet and OS (**A**) High tumor islet CD68^+^ TAM density predicted better OS (HR = 0.50, 95% CI = 0.30 ~ 0.85, *P* = 0.01; I^2^ = 83%, *P* = 0.0005). (**B**) High tumor islet M1 TAM density was associated with better OS (HR = 0.23, 95% CI = 0.18 ~ 0.29, *P* < 0.00001; I^2^ = 0%, *P* = 0.49). (**C**) Tumor islet M2 TAM density was not associated with OS (HR = 0.83, 95% CI = 0.43 ~ 1.579, *P* = 0.56; I^2^ = 71%, *P* = 0.03).

The micro-distribution of TAMs in the tumor microenvironment may play a role in lung cancer progression according to the included studies. Four publications reported the association between islet CD68^+^ TAMs and OS (Table 1). The pooled HR showed that high density of CD68^+^ cells in the tumor islet predicted better OS (HR = 0.50, 95% CI = 0.30 ~ 0.85, *P* = 0.01; I^2^ = 83%, *P* = 0.0005; Figure 3A). As for different TAM polarizations in tumor islet, high density of CD68^+^HLA-DR^+^ M1 TAMs was also associated with better OS (HR = 0.23, 95% CI = 0.18 ~ 0.29, *P* < 0.00001; I^2^ = 0%, *P* = 0.49; Figure 3B), while islet CD68^+^CD163^+^ M2 density was not associated with OS (HR = 0.83, 95% CI = 0.43 ~ 1.579, *P* = 0.56; I^2^ = 71%, *P* = 0.03; Figure 3C).

### Stromal TAM density and OS

**Figure 4:**
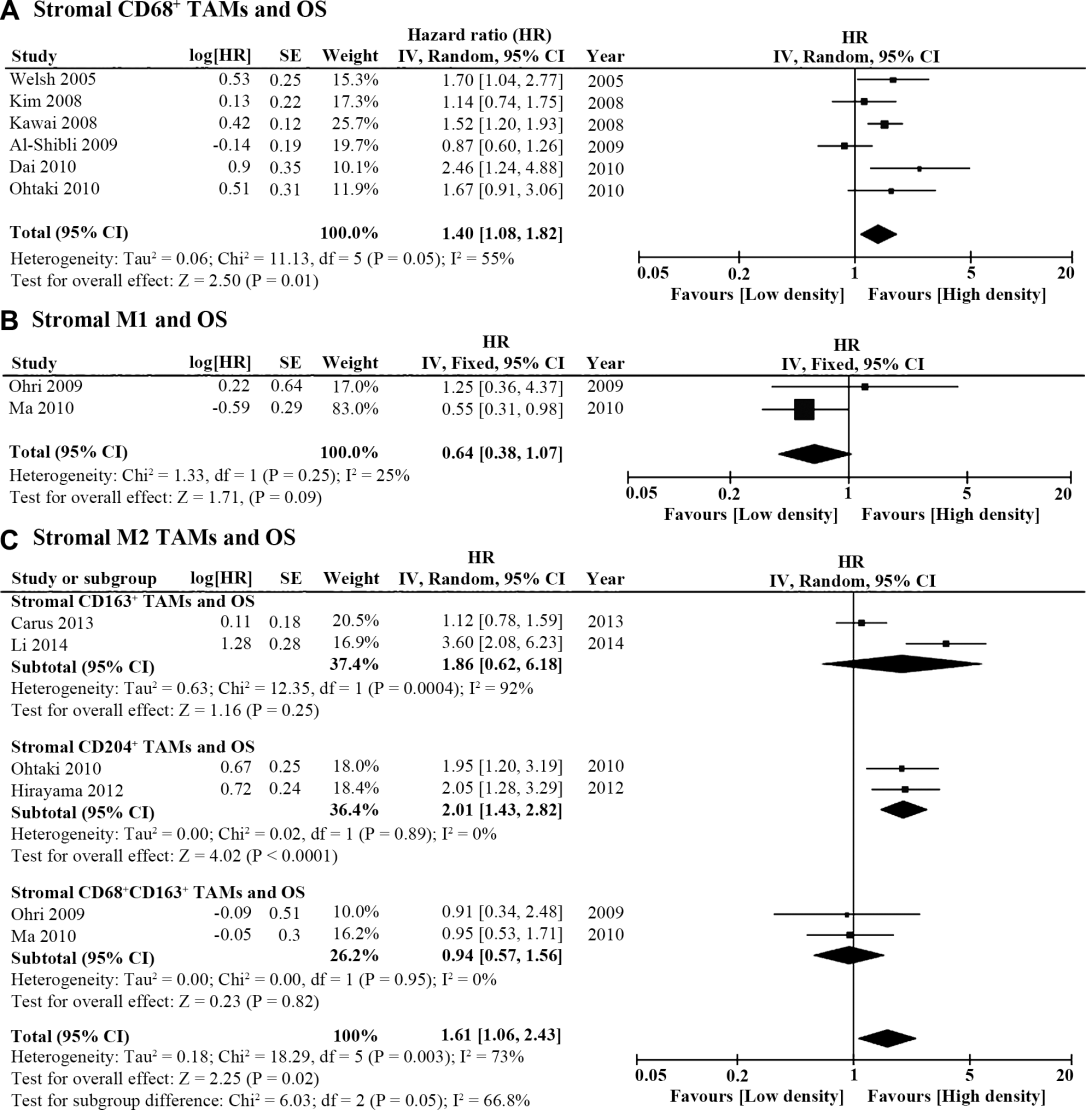
Forest plot of HR for TAM density in the tumor stroma and OS (**A**) High stromal CD68^+^ TAM density was associated with poor OS (HR = 1.40, 95% CI = 1.08 ~ 1.82; *P* = 0.01; I^2^ = 55%, *P* = 0.05). (**B**) Stromal M1 TAM density was not associated with OS (HR = 0.64, 95% CI = 0.38 ~ 1.07, *P* = 0.09; I^2^ = 25%, *P* = 0.25). (**C**) High density of M2 TAMs (using different markers) in the tumor stroma was associated with poor OS (HR = 1.61, 95% CI = 1.06 ~ 2.43, *P* = 0.02; I^2^ = 73%, *P* = 0.003). However, a subgroup analysis revealed that only high density of CD204^+^ M2 TAMs was associated with poor OS (HR = 2.01, 95% CI = 1.43 ~ 2.82, *P* < 0.0001; I^2^ = 0%, *P* = 0.89).

Six studies reported stromal TAM density and OS (Table 1). The pooled HR of these studies revealed that high stromal CD68^+^ cell count was associated with worse OS (HR = 1.40, 95% CI = 1.08 ~ 1.82; *P* = 0.01; I^2^ = 55%, *P* = 0.05; Figure 4A). In addition, CD68^+^HLA-DR^+^ M1 polarization was not associated with OS according to the pooled HR of 2 studies (HR = 0.64, 95% CI = 0.38 ~ 1.07, *P* = 0.09; I^2^ = 25%, *P* = 0.25; Figure 4B). As for the study of stromal M2 density, different markers and staining techniques were applied in 6 studies. Two groups studied stromal M2 density with double staining of CD68 and CD163. The remaining 4 studies used either CD163 or CD204 as M2 markers in each of the 2 publications. The combined HR of these 6 studies showed high density of M2 TAMs in the stroma was associated with poor OS (HR = 1.61, 95% CI = 1.06 ~ 2.43, *P* = 0.02; I^2^ = 73%, *P* = 0.003; Figure 4C). Subgroup analysis was conducted to pool studies using different M2 markers. Only stromal CD204^+^ M2 TAM density was associated with OS (high density vs. low density, HR = 2.01, 95% CI = 1.43 ~ 2.82, *P* < 0.0001; I^2^ = 0%, *P* = 0.89; Figure 4C).

### Islet/stromal (I/S) ratio of TAM density and OS

**Figure 5:**
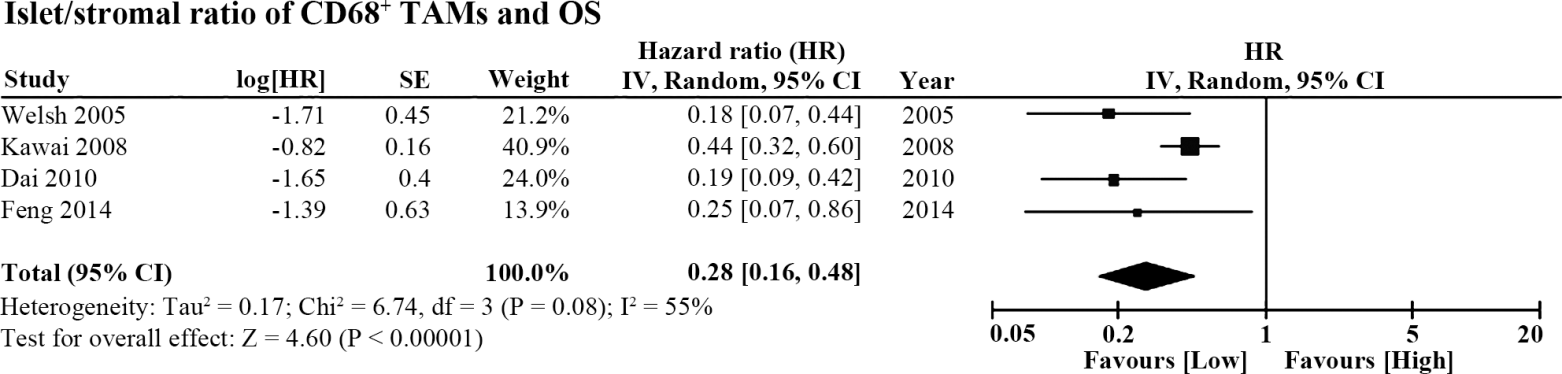
Forest plot of HR for the islet/stromal (I/S) ratio of CD68^+^ TAM density and OS Higher I/S ratio of CD68^+^ cell number was associated with better OS in lung cancer patients (HR = 0.28, 95% CI = 0.16 ~ 0.48, *P* < 0.00001; I^2^ = 55%, *P* = 0.08).

Four of the 20 studies also reported the association between the ratio of islet/stromal (I/S) CD68^+^ TAM density and OS. The pooled HR of these 4 studies showed that higher I/S ratio of CD68^+^ TAMs also indicated better OS in patients with NSCLC (HR = 0.28, 95% CI = 0.16 ~ 0.48, *P* < 0.00001; I^2^ = 55%, *P* = 0.08; Figure 5).

### Correlation between TAM infiltration and clinicopathological characteristics

Some of the publications reported the association between TAM density and clinicopathological characteristics. We focused on the association between TAM density and patient demographic characteristics as well as TNM stage if the data were available for different TAM markers or micro-distribution. Both CD68^+^ TAM density and micro-distribution (islet and/or stroma) in lung cancer tissues were studied. The main results of TAM infiltration and patient characteristics were summarized in Table 2. There were no association between the I+S CD68^+^ TAM density and gender (male vs. female), age (> 60 vs. < 60 years old), histologic type (ADC vs. non-ADC) or TNM stage (III–IV vs. I–II). As for the islet CD68^+^ TAMs, there was no difference between different histologic types between the low and high TAM density groups. However, there were more stage III-IV patients in the low islet CD68^+^ TAM group (OR = 0.52, 95% CI = 0.34 ~ 0.81, *P* = 0.004; I^2^ = 0%, P = 0.82). In addition, high stromal CD68^+^ TAM density was associated with male gender (OR = 2.22, 95% CI = 1.37 ~ 3.60,*P* = 0.001; I^2^ = 0%, *P* = 0.82), poor differentiation (OR = 2.93, 95% CI = 1.14 ~ 7.56, *P* = 0.03; I^2^ = 81%, *P* = 0.006), and advanced lymph node stage (N-stage; OR = 2.45, 95% CI = 1.41 ~ 4.26, *P* = 0.002; I^2^ = 0%, *P* = 0.42). There were 2 studies that evaluated stromal CD204^+^ TAMs and patient characteristics. The pooled HR revealed that high stromal CD204^+^ TAM density was associated with advanced tumor stage (T-stage; OR = 2.83, 95% CI = 1.52 ~ 3.72, *P* = 0.0002; I^2^ = 0%, *P* = 0.70).

**Table 2:** The relationship between TAMs and clinicopathological characteristics

Patient characteristics	Studies (Ref No.)	Overall OR (95% CI)	Heterogeneity test			*p*-value
			Chi^2^	I^2^	*p*-value	
**CD68^+^ TAMs (I+S) and clinicopathological characteristics**						
Gender (Male vs. Female)	[15, 23]	1.38 (0.86, 2.20)	0.43	0%	0.51	0.18
Age (> 60 vs. < 60 years)	[15, 23]	0.64 (0.13, 3.29)	2.73	63%	0.10	0.60
Histology (ADC vs. Non-ADC)	[15, 18, 23]	1.47 (0.64–3.39)	4.83	59%	0.09	0.37
p-stage (III–IV vs I–II)	[15, 23]	1.23 (0.80, 1.90)	0.51	0%	0.48	0.35
**Islet CD68^+^ TAMs and clinicopathological characteristics**						
Histology (ADC vs. Non-ADC)	[18, 29]	1.09 (0.48, 2.49)	2.55	61%	0.11	0.84
p-stage (III–IV vs I–II)	[18, 29, 32]	0.52 (0.34, 0.81)	0.41	0%	0.82	0.004
**Stromal CD68^+^ TAMs and clinicopathological characteristics**						
Gender (Male vs. Female)	[26, 29]	2.22 (1.37, 3.60)	0.05	0%	0.82	0.001
Smoker (Yes vs. No)	[26, 29]	2.16 (0.81, 5.76)	4.27	77%	0.04	0.13
Histology (ADC vs. Non-ADC)	[18, 29, 32]	1.50 (0.69, 3.28)	7.10	72%	0.03	0.30
Grade (poor vs. well)	[18, 29, 32]	2.93 (1.14, 7.56)	10.3	81%	0.006	0.03
N-stage (N_1–2_vs N_0_)	[18, 26]	2.45 (1.41, 4.26)	0.66	0%	0.42	0.002
p-stage (III–IV vs I–II)	[18, 29]	1.17 (0.48, 2.83)	2.64	62%	0.10	0.73
**Stromal CD204^+^ TAMs and clinicopathological characteristics**						
Gender (Male vs. Female)	[17, 26]	1.81 (0.40, 8.12)	7.02	86%	0.008	0.44
Age (> 70 vs. < 70 yrs)	[17, 26]	0.82 (0.54, 1.26)	0.40	0%	0.53	0.36
Smoker (Yes vs. No)	[17, 26]	1.50 (0.09, 23.81)	14.0	93%	0.002	0.77
T-stage (T_1_ vs T_2–4_)	[17, 26]	2.83 (1.52, 3.72)	0.15	0%	0.70	0.0002
N-stage (N_1–2_vs N_0_)	[17, 26]	4.33 (0.96, 19.62)	6.90	86%	0.009	0.06

### Publication bias

**Figure 6:**
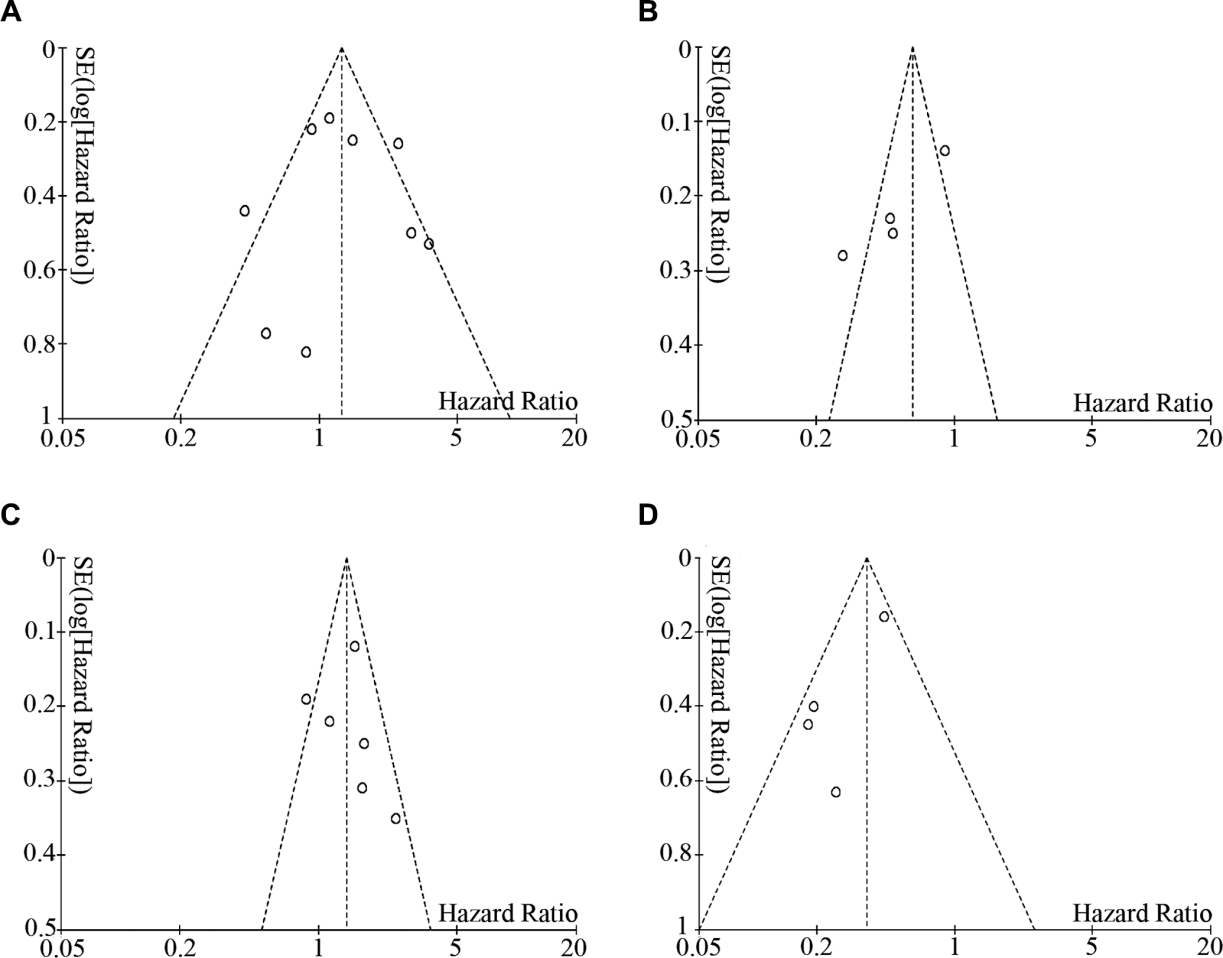
Begg's funnel plot showed no publication bias among the included studies (**A**) I+S CD68^+^ TAMs and OS (*P* = 0.677). (**B**) Islet CD68^+^ TAMs and OS (*P* = 0.174). (**C**) Stromal CD68^+^ TAMs and OS (*P* = 0.348). (**D**) I/S ratio of CD68^+^ TAMs and OS (*P* = 1.000).

Begg's funnel plot and Egger's test were employed to investigate publication bias among the included studies on CD68^+^ TAMs and OS. Both tests indicated no publication bias among the studies regarding the I+S CD68^+^ TAMs and OS (Begg's test, *P* = 0.677, Figure 6A; Egger's test, *P* = 0.951). As for the studies of the islet CD68^+^ TAMs and OS, Begg's test showed no publication bias (*P* = 0.174, Figure 6B), whereas Egger's test revealed statistical significance (*P* = 0.027) among the studies. The studies about stromal CD68^+^ TAMs and OS were also evaluated with Begg's and Egger's tests and showed no evidence of publication bias (Begg's test, *P* = 0.348, Figure 6C; Egger's test, *P* = 0.700). There was no evidence of publication bias among the studies regarding the I/S ratio of CD68^+^ TAMs and OS (Begg's test, *P* = 1.000, Figure 6D; Egger's test, *P* = 0.105). Due to the small number of studies focused on TAM polarization or micro-distribution, the tests for publication bias were not performed.

## DISCUSSION

The treatment of lung cancer remains a thorny problem, especially for those with advanced stage diseases. Surgery is the most preferred treatment option for patients with early stage NSCLC [37, 38]. For patients with more advanced diseases, the widely accepted therapeutic strategies are chemotherapy and/or radiotherapy, while targeted therapy is an alternative for some patients with sensitive mutations [38]. Recently, immunotherapy emerged as a therapeutic promise for patients with different kinds of cancers, including NSCLC [3]. Comprehensive understanding of tumor immune microenvironment may contribute to the development of novel immunotherapeutic agents.

TAMs are important component of tumor immune microenvironment and promote tumor progression via various mechanisms, including promoting therapeutic resistance, angiogenesis, immune suppression and metastasis [7]. The multifunctional properties of TAMs in tumor progression indicate that targeting this group of immune cells may represent a novel immunotherapeutic strategy. Using a subcutaneous lung cancer model, Ren et al. found that interferon-γ and/or celecoxib attenuated tumor growth through modulating M2/M1 TAM ratio [39]. Overexpression of Fpr2 gene favored M1 polarization and inhibited the growth of subcutaneously implanted Lewis lung cancer [40]. In addition, targeting TAMs with anti-colony-stimulating factor 1 receptor (CSF-1R) monoclonal antibody RG7155 resulted in depletion of TAMs and objective response of the tumor [41]. Based on the pre-clinical studies, multiple clinical trials are ongoing to validate the anti-tumor effects of different agents targeting macrophage recruitment, polarization, function and activation [7].

The role of TAMs varies among different solid tumors in multiple studies performed with human samples. High density of TAMs was associated with worse survival in gastric cancer and head and neck cancer, but was associated with better OS in patients with colorectal cancer [42]. Nevertheless, there are conflicting data among the studies of TAMs and their micro-distribution in lung cancer patients [15–19, 27, 32]. In order to reveal the potential role of TAMs in NSCLC prognosis, we conducted this meta-analysis to evaluate TAM infiltration and patient survival based on different markers and micro-distribution in lung cancer tissues. The results showed that neither I+S CD68^+^ TAM density nor CD68^+^CD163^+^ M2 TAM density was correlated with survival. However, low I+S density of CD68^+^HLA-DR^+^ M1 TAMs was found to be associated with poor OS. As for the micro-distribution of TAMs and survival, low density of CD68^+^ TAMs and CD68^+^HLA-DR^+^ M1 TAMs in lung cancer islet were both associated with poor OS, while islet CD68^+^CD163^+^ M2 TAM density was not correlated with prognosis. High density of stromal CD68^+^ TAMs or CD204^+^ M2 TAMs were associated with poor OS, whereas stromal CD68^+^HLA-DR^+^ M1 and CD68^+^CD163^+^ M2 TAM densities were irrelevant to OS. Low ratio of islet to stromal (I/S) CD68^+^ TAM density also predicted poor OS.

CD68 is the most commonly used marker for the study of TAMs. A total of 12 out of the 20 included studies used CD68 as macrophage marker [15, 18, 23, 24, 28–30, 32–36], while the other 4 studies used CD68 in combination with other markers for the detection of TAMs [19, 25–27]. Unlike some other solid tumors, the total number of I+S CD68^+^ TAMs was not associated with survival in lung cancer patients, while low islet and high stromal CD68^+^ TAMs were both associated with poor OS. This may be partially due to the reverse prognostic impacts of CD68^+^ TAMs in lung tumor islet and stroma. In addition, Gottfreid and colleagues studied the expression of CD68 in different primary cells and cancer cell lines, and found that CD68 expression was widespread, including monocytes, macrophages, fibroblasts, endothelial cells and even some cancer cells [43]. CD68 may not be a specific marker of macrophages but only enriched in this group of cells [43]. Therefore, the use of CD68 as the only macrophage marker may have some confounding effects.

In addition, several studies focused on the tumor-promoting M2 phenotype using more specific markers, including CD163 and CD204, or double staining of CD68 and CD163. Though the pooled HR of these studies confirmed the role of M2 TAMs in tumor progression, the prognostic role of different M2 markers varied according to subgroup analysis. Neither CD68^+^CD163^+^ TAMs nor CD163^+^ TAMs were associated with patient survival. Only CD204^+^ TAM density was found to be associated with OS. However, this should be further validated with larger samples.

Opposite survival effects of CD68^+^ TAM infiltration in the tumor islet and stroma were observed in lung cancer patients. High islet CD68^+^ TAM density showed similar effects as high islet M1 infiltration which was associated with better survival. However, high stromal CD68^+^ TAM density was related to poor OS, similar to high stromal M2 TAM infiltration. Ohri et al. demonstrated that 70% of the TAMs in the lung cancer islet were M1 type [19]. In addition, the proportion of M2 TAMs in the lung cancer stroma was much higher than that of M1 [27]. The differences in the distribution of M1 and M2 TAMs were consistent with the survival data, reflecting their anti- and pro-tumor functions. The opposite polarization of CD68^+^ TAMs in the tumor islet and stroma may also interpret the different effects of these cells on patient survival.

In these published reports, only a small number of the studies described the association between TAM infiltration and patient characteristics. There was no relationship between I+S CD68^+^ TAM density and clinicopathological features, including gender, age, histologic type and pathological stage. However, low islet CD68^+^ TAM density was found to be associated with more advanced pathological stage, while high stromal CD68^+^ TAM density was relevant to male gender, poor differentiation, and the presence of lymph node metastasis. Furthermore, high stromal CD204^+^ M2 TAM density was found to be associated with more advanced T-stage. Both islet and stromal CD68^+^ TAM density and stromal CD204^+^ M2 TAM density were associated with patient survival. Due to the limited data available, the stratified analysis to evaluate the effects of stage on TAM infiltration and patient survival was not performed.

High stromal CD68^+^ TAM infiltration was found to be associated with male patients. However, gender was not an independent prognostic factor according to the multivariate analysis in different studies [15, 17, 27, 29]. The exact implication of the higher stromal CD68^+^ TAM density in male patients remains unknown. Nevertheless, gender should be taken into consideration when selecting patients for future clinical trials.

Though we tried our best to perform a comprehensive analysis among TAM polarization, micro-distribution and patient survival, there were still some shortcomings of the present study. First, the heterogeneity tests were significant in some of the pooled HRs of OS. The potential causes to explain the heterogeneity included the antibodies or methodologies used in different studies, the histologic type, origin of the patients, different edition of TNM staging system applied, and potential publication bias. Second, there was a potential risk of language bias since we only included the publications in English and Chinese. Third, some of the HRs with 95% CIs were extracted from the Kaplan-Meier survival curve. Tumor stage is an important prognostic factor. The differences in tumor stages between the high and low TAM density groups may be a confounding factor for the observation of OS. Fourth, the number of the included studies was relatively small.

In conclusion, the present meta-analysis confirmed the potential prognostic role of TAMs in NSCLC patients. Though the total count of CD68^+^ TAMs was not a prognostic factor, CD68^+^ TAM density in the lung cancer islet and stroma were both associated with patient survival. Low islet and high stromal CD68^+^ TAM density predicted poor survival. Low islet M1 count or high stromal M2 count also showed potential association with tumor progression. Further studies with standardized methodology and larger sample size are warranted to validate the conclusions.

## MATERIALS AND METHODS

### Literature searching strategy

We performed electronic literature searches using PubMed, Embase, Cochrane library and Web of Science on April 16th, 2015. Studies published between January 1996 and April 2015 were selected using the following searching strategy: “lung cancer” or “lung carcinoma” or “lung neoplasm” and “Macrophage”. The references of the identified articles were also reviewed to identify potentially relevant articles.

### Study selection

All of the eligible articles focusing on the prognostic role of macrophages in primary NSCLC were included in this meta-analysis according to the following criteria: (1) macrophage density evaluated in primary NSCLC; (2) macrophage infiltration in NSCLC was described as high (above the cut-off value or positive) and low (below the cut-off value or negative) density; (3) overall survival (OS) and/or disease-free survival (DFS) were analyzed. The exclusion criteria were: duplicate reports, case reports, reviews, conference abstracts, *in vitro* or animal studies, non-English/non-Chinese publications, studies with duplicate cases, and studies with insufficient data for the evaluation of hazard ratio (HR) and 95% confidence interval (CI) about OS and/or DFS. If the publications studied the same group of patients using different macrophage markers, all of them were included for marker-specific analysis.

### Assessment of study quality and data extraction

Two researchers (J. Mei and Z. Xiao) independently reviewed and evaluated the included studies using the Newcastle-Ottawa Scale (NOS) [20]. Disagreement between the researchers was resolved by discussion with a third researcher. After reviewing the full text, two researchers extracted the data, including surname of the first author, publication year, origin of the study, study period, sample size, histologic type, stage, macrophage markers, grouping method, the relationship between TAM distribution and/or polarization. The HR and 95% CI were calculated with the data extracted from survival curves, using the method described by Tierney et al. [21]. Engauge Digitizer 4.1 (http://digitizer.sourceforge.net) was used for data extraction from the survival curves.

### Statistical analysis

Data analysis was carried out using Review Manager 5.3 (Cochrane Collaboration, Oxford, UK) and Stata 12.0 (Stata Corporation, Texas, US). HR with 95% CI was used to assess the significance of TAM density on OS and DFS of the patients with NSCLC. Odds ratio (OR) was pooled by Mantel-Haenszel method to estimate the relationship between TAM density and clinicopathological characteristics. Heterogeneity of the included studies was tested by Chi-square test with *p*-value set at less than 0.10. I-square (I^2^) test was applied to assess total variation among the studies. If *p* < 0.10 or I^2^ > 50%, the random effect model was applied to pool the data; otherwise, we chose the fixed effect model. Potential publication bias was assessed using Egger's test and Begg's test.
